# Functional status after major amputation in diabetic foot patients from a tertiary hospital

**DOI:** 10.1186/1758-5996-7-S1-A21

**Published:** 2015-11-11

**Authors:** Ligia de Loiola Cisneros, Aline da Conceição Arruda, Tulio Pinho Navarro

**Affiliations:** 1 Universidade Federal de Minas Gerais, Belo Horizonte, Brazil

## Background

The impact of global diabetes burden is evidenced by the growing morbidity and mortality rates, and by permanent disabilities such as blindness, diabetic retinopathy, end-stage renal failure and lower extremity amputations.

## Objective

The purpose of this study was to verify functional status of diabetic foot patients submitted to lower limb major amputation after the discharge of a university hospital, reference in vascular surgery. A cohort of 31 diabetic foot patients was identified from the electronic medical records. Data were retrospectively collected from each patients chart including age, gender, peripheral arterial disease, level of amputation and number of readmissions. Patients were contacted by phone. A questionnaire investigated the survival, functional and ambulatory status: prosthesis use, reason for non-implantation of prosthetics, other hospitalization and amputation, mobility, self-performance of activities of daily living such as dressing and personal hygiene.

## Results

The mean age of patients at the time of surgery was 65.23 yrs.(SD 11.86), 21 (67.7%) patients were men. Peripheral arterial disease was present in 17 (54.8%) of the sample. Below-the-knee amputation was performed in 16 (51.6%), the mean time of amputation was 48.71 months (SD 21.29). From the total, 15 (48.4%), were admitted only once and 6 (19.4%) were readmitted one more time for diabetic foot problems and 4 (12.9%) were submitted to other amputation, 12 (38.8%) died. A total of 15 (48.4%) subjects were fitted with prosthesis. Others diabetes complications were related by 5 (16.1%) patients as the main reason for non-implantation of prosthetics. Twelve (38.8%) patients were able to walk, 15 (48.4%) were on wheelchair and 4 (12.8%) rest on bed after the amputation. A reduction in self-performance of activities of daily living were reported by 11 (35.5%) patients.

## Conclusion

These findings exemplify the decrements in functioning for elderly diabetic foot patients after a major amputation.

